# Elderly persons in the risk zone. Design of a multidimensional, health-promoting, randomised three-armed controlled trial for "prefrail" people of 80+ years living at home

**DOI:** 10.1186/1471-2318-10-27

**Published:** 2010-05-26

**Authors:** Synneve Dahlin-Ivanoff, Gunilla Gosman--Hedström, Anna-Karin Edberg, Katarina Wilhelmson, Kajsa Eklund, Anna Duner, Lena Ziden, Anna-Karin Welmer, Sten Landahl

**Affiliations:** 1 Vårdalinstitutet, The Swedish Institute for Health Sciences, Universities of Gothenburg and Lund, Sweden; 2 Department of Clinical Neuroscience and Rehabilitation, The Sahlgrenska Academy at University of Gothenburg, Gothenburg, Sweden; 3 Department of Health Sciences, Lund University, Lund, Sweden; 4 Department of Public Health and Community Medicine/Social Medicine, Institute of Medicine, The Sahlgrenska Academy at University of Gothenburg, Gothenburg, Sweden; 5 Department of Social Work, University of Gothenburg, Gothenburg, Sweden; 6 Karolinska University Hospital, Stockholm, Sweden

## Abstract

**Background:**

The very old (80+) are often described as a "frail" group that is particularly exposed to diseases and functional disability. They are at great risk of losing the ability to manage their activities of daily living independently. A health-promoting intervention programme might prevent or delay dependence in activities of daily life and the development of functional decline. Studies have shown that those who benefit most from a health-promoting and disease-preventive programme are persons with no, or discrete, activity restrictions. The three-armed study "Elderly in the risk zone" is designed to evaluate if multi-dimensional and multi-professional educational senior meetings are more effective than preventive home visits, and if it is possible to prevent or delay deterioration if an intervention is made when the persons are not so frail. In this paper the study design, the intervention and the outcome measures as well as the baseline characteristics of the study participants are presented.

**Methods/Design:**

The study is a randomised three-armed single-blind controlled trial with follow-ups 3 months, 1 and 2 years. The study group should comprise a representative sample of pre-frail 80-year old persons still living at home in two municipalities of Gothenburg. To allow for drop-outs, it was estimated that a total of about 450 persons would need to be included in the study. The participants should live in their ordinary housing and not be dependent on the municipal home help service or care. Further, they should be independent of help from another person in activities of daily living and be cognitively intact, having a score of 25 or higher as assessed with the Mini Mental State Examination (MMSE).

**Discussion:**

We believe that the design of the study, the randomisation procedure, outcome measurements and the study protocol meetings should ensure the quality of the study. Furthermore, the multi-dimensionality of the intervention, the involvement of both the professionals and the senior citizens in the planning of the intervention should have the potential to effectively target the heterogeneous needs of the elderly.

**Trial registration:**

ClinicalTrials.gov, NCT00877058

## Background

The very old are often described as a "frail" group that is particularly exposed to diseases and functional disability and who are at great risk of losing their ability to manage their activities of daily living independently [1-4]. It is a heterogeneous group, consuming a large proportion of the resources of both the health and the care and special services. It is also a group whose care, special service and rehabilitation needs vary considerably and change rapidly [5,6], requiring different professional contributions to be able to offer as adequate and appropriate intervention as possible. The ageing population is posing a challenge to the Swedish welfare system, which will increase future costs for elderly care and health care. The parliamentary bill [7]points out that research will be essential to support the development of effective and evidence-based methods of a preventive and health-promoting nature. Directing efforts for elderly men and women can lead to great gains, both health-wise and economically, both for the individual and society at large [7]. Thus, in the area of public health research, priority should be given to randomised studies evaluating the effect of such interventions in order to be able to meet the challenge posed by the growing proportion of elderly persons in the general population and the possible increasing need for health care this could generate. In this paper, we describe the design of a health-promoting and preventive intervention study directed to elderly "prefrail" people above the age of 80.

Frailty has become an established concept in research in recent years [1,8,9]. A state of decreased reserve resistance to stressors as a result of cumulative decline across multiple physiological systems is a common definition of frailty [1]. Frailty can range from mild to severe stages, and it is regarded as being strongly linked to restricted activity and morbidity. The concept [1], usually includes weakness, fatigue, weight loss, low physical activity, poor balance, slow gait speed and impaired cognition. Visual impairment is not usually included, but in recent years it has been highlighted as an important indicator of frailty, based on its impact on morbidity and disability [10-12].

The ability to manage activities of daily life deteriorates with age, and early signs such as experienced difficulties, insecurity and fatigue are often followed by the need of assistance from someone else in order to manage daily activities [13-15]. A health-promoting intervention programme could prevent and delay dependence in activities of daily life as well as the development of functional decline. Literature reviews [16-18] have examined interventions based on preventive home visits. Van Haastregt et al. [18] examined 15 trials of preventive home visits to elderly people and concluded that there was no clear evidence that preventive home visits to elderly people living in the community were effective. Stuck et al. [18]examined 18 trials, and their results indicated that home visits were effective if they were multi-dimensional and included several follow-up visits addressing "younger elderly" whose health had not yet been markedly affected. A follow-up meta-analysis [16] examining the effect of home visits to elderly (70+) persons living at home concluded that home visits reduced the disability burden among older adults if they were based on multidimensional assessment, including clinical examination. Furthermore, other studies have confirmed that those who probably benefit most from a health-promoting and preventive programme are people who have not yet suffered any restriction in activity levels or those in the early stages of activity restriction [8,19,20].

A recent review that included 14 randomised controlled studies (RCT) of multi-dimensional programs focusing specifically on frail elderly persons [21] found it difficult to conclude whether some intervention components were more successful than others, and if working in a interdisciplinary team made any difference to the outcome. The authors declared though that a multi-dimensional intervention programme targeting frail elderly persons needs diverse professionals to be able to offer a broad spectrum of intervention components to carry out an effective programme. They concluded that different professionals most likely had their own ways of "framing the problem", which implies that an interdisciplinary intervention team is desirable when dealing with the complexity of frailty. Another review [22] investigating the benefit of multidisciplinary teamwork targeting frail elderly persons living in the community concluded that our knowledge regarding the impact of multidisciplinary teams working with elderly persons is still limited.

In summary, findings from the above-mentioned studies guided the design of the health-promoting and multi-dimensional as well as multi-professional intervention named" Elderly persons in the risk zone". The study addresses very old (80+) elderly persons that are on the point of developing frailty ("pre-frail"). As the intervention is complex, both quantitative and qualitative methods were required to capture the multidimensionality of the provision and the different effects of the study. It was hoped that giving the respondents opportunities to highlight their individual priorities in their own ways might reveal ideas that had not been anticipated by the researchers, and also improve our understanding of the impact of the intervention [23]. Furthermore, the complex factors that affect the habits and ways of thinking about health and illness will be illuminated. Knowing more about how the "pre-frail" elderly persons themselves understand their health will enhance the results of the intervention study and contribute to the development of high quality care and support for this group.

### Aims and hypotheses

The authors of this intervention study aimed to prove the following two hypotheses:

1) If an intervention is made when the elderly persons are not so frail, it is possible to prevent/delay deterioration;

2) A multi-dimensional and multi-professional intervention is more effective than preventive home visits alone.

This paper presents the study design, the intervention and the outcome measures as well as the baseline characteristics of the study participants in accordance with the CONSORT recommendations for reporting pragmatic RCT [24].

## Methods/Design

### Project context

The present study was conducted in two municipalities in Gothenburg, Sweden, in which people over 80 years of age account for 8 and 7% respectively of the population, compared to Gothenburg as a whole (5%) and Sweden as a whole (5%). The aim of the municipal provision of care for the elderly is to ensure that these persons are able to live as independent lives as possible. This includes living in their own homes. When an elderly person in Sweden is no longer able to manage independently, she or he can apply for assistance from the municipal home-help services. The extent of such support is subject to an assessment of needs. The support includes meals on wheels, help with cleaning and shopping, assistance with personal care, safety alarms as well as transportation service. The elderly are also offered health care, provided either by the municipal home-help services or by the home medical care service.

### Study design

The study has an explorative, descriptive, analytical and experimental design with a follow-up for two years. The participants were randomised to three study arms: two intervention groups and one control group. Focus groups and individual in-depth interviews were conducted so that the researchers could gain an understanding of the intervention and its significance. Ethical approval was obtained for the study "Elderly persons in the risk zone" ref. no: 650-07.

### Study population

The intention was that the study group should comprise a representative sample of pre-frail 80-year old persons still living at home in two municipalities of Gothenburg. Criteria for inclusion: The participants should live in their ordinary housing and not be dependent on the municipal home help service or care. Further, they should be independent of help from another person in activities of daily living and be cognitively intact, having a score of 25 or higher as assessed with the Mini Mental State Examination (MMSE).

### Intervention

#### Intervention A; Senior meetings and one follow-up home visit

This intervention comprised 4 weekly educational senior meetings with no more than 6 participants in each group. The main purpose was to focus on two different areas, 1) information about the ageing process and its consequences, and 2) providing tools and strategies for solving the various problems that may arise in the home environment. A follow-up home visit took place about 2-3 weeks after the group had completed the group education. The group meetings were led either by an occupational therapist, a registered nurse, a physiotherapist or a qualified social worker, who jointly planned and carried out the intervention and had responsibility for their specific part of the education. A booklet especially designed for the study group was used as a basis for the meetings. The book includes texts, for example, about self-care strategies and information referring to the topics discussed at each meeting. The booklet was thus an important educational tool [25].

#### Intervention B; Preventive home visit

This intervention was in the form of a single home visit made by a nurse, a physiotherapist, a qualified social worker or an occupational therapist. During this visit the clients received verbal and written information and advice about what the municipality could provide in the form of local meeting places, activities run by local associations, physical training for seniors, walking groups etc. The clients were also informed about help and support of various kinds offered either by volunteers or by professionals employed by the municipality. They were also informed about assistive devices and adaptation of housing. Fall risks were also identified and advice on how to prevent falls were also included in the home visit. Information was also given about who they could contact for different problems.

#### Control group

This group had access to the ordinary range of services from the municipal care for the aged. If the investigator discovered that a person in the control group had any kind of need, he/she was informed about where to go for problems concerning municipal aged care.

### Research questions and outcome measurements

1. Can a health-promoting and preventive intervention for "prefrail" elderly persons:

• prevent frailty, activity limitations and morbidity,

• be a supportive factor in the social and physical environment,

• affect life satisfaction

• have an impact on the consumption of care

• be cost-effective?

2. How do the frail elderly persons experience the intervention and its importance to health?

#### Primary Outcome Measures

Frailty indicators (weakness, fatigue, weight loss, low physical activity, poor balance, slow gait speed and impaired cognition), performance of daily activities and morbidity.

#### Secondary Outcome Measures

Quality of life, life satisfaction, assistive technology, accessibility, feeling of loneliness, social interaction, social support, participatory activities, falls, fear of falling, health care consumption and mortality.

#### Measurements of frailty indicators

##### Weakness

Grip strength was measured using a North Coast dynamometer [26]. The starting position was sitting on a chair comfortably. The shoulder should be adducted and neutrally rotated. The elbow should be flexed to 90 degrees. The forearm and wrist should be in a neutral position. The dynamometer was set to mode 2, as recommended in the manual. Measurements were carried out three times per hand, with a rest in between, starting with the dominant hand. The maximum value in the dominant hand was used. In this study, reduced strength was considered to be below 13 kg for women and 21 kg for males for the right hand, and below 10 kg for women and 18 kg for males for the left hand.

##### Fatigue

The subject was asked the following question: "Have you suffered any general fatigue/tiredness over the last three months?" This question is listed under the symptoms measured with "The Göteborg quality of life instrument[27], which is a self-estimate tool giving reliable and stable measurements of symptoms. In this study those who answered yes were classified as frail.

##### Weight loss

The subject was asked the following question; "Have you suffered from any weight loss over the last three months?" This question is listed under the symptoms measured with "The Göteborg quality of life instrument (GQL) [27], which is a self-estimate tool giving reliable and stable measurements of symptoms. In this study those who answered yes were classified as frail.

##### Physical activity

This was measured with the help of a six-point scale on which the participants recorded how often they took outdoor walks. In this study 1-2 walks/week or less was considered to be reduced physical activity, and the participants who recorded this score were classified as frail.

##### Balance

This was measured with the Berg Balance Scale (BBS) [28,29] The instrument measures balance in 14 items and the assessment is made by observation. Every moment is scored using a 5-point scale (0-4). The instrument can be used on both individual and group level and has been tested for validity, reliability and sensitivity. The maximum score is 56 points. In this study, a value of 47 or lower was classified as a frailty indicator [30].

##### Gait speed

Walking four metres at a comfortable speed was taken as a measure of gait speed. If the best speed value was 0.6 metres per second or slower, this was considered to be an indicator of frailty [31].

##### Visual impairment

The KM chart [32] is a letter chart adjusted for one metre distance that measures visual acuity from 0.1-1.0. The visual acuity recorded was when 70% of the letters of the current line were correctly identified, corresponding to clinical practice. If the participant had their own glasses, they were used at the time of the examination. In this study a visual acuity of ≤ 0.5 in both eyes was classified as visual impairment.

##### Impaired cognition

This was measured with the Mini Mental State Examination (MMSE) [33]. For inclusion in the study, the participants had to be cognitively intact as assessed with a MMSE, and a cut-off of below 25 was used longitudinally to identify frailty at follow-ups.

#### Economic evaluation

The economic analysis of the intervention study will be made in the form of a cost-utility analysis (CUA), which is a variant of cost-effectiveness analysis. In a CUA the health effects of the intervention are quantified as quality-adjusted life years (QALY). The main outcome of the CUA is the incremental costs per QALY. The incremental cost utility ratio (ICUR) is calculated by comparing the difference between the intervention and the control groups in average costs per person to the difference in QALY per person. Data relating to health-related quality of life (HRQL) will be collected at baseline, 3 months, 1 and 2 years. The QALYs of the three groups will be estimated by calculating the area under the curve. Various perspectives can be taken when conducting economic analyses, such as the perspective of the hospital, primary payer or society. The perspective of this study is that of society. This implies that an attempt will be made to account for the consequences of all resource use. The costs included constitute intervention costs, and the health- and home care sector.

Table 1 gives a description of the objectives, outcome measurements and follow-ups in the study

**Table 1:** Outcome measurements and follow-ups

Primary Outcome	Measurement	TO	T1	T2	T3
			3 month	1 year	2 year
Fatigue	Questionnaire/tiredness scale	X	X	X	X
Grip strength	North Coast dynamometer	X		X	X
Endurance/physical activity	Questionnaire/physical and	X	X	X	X
	activity scale	X	X	X	X
Balance	The Berg Balance Scale	X	X	X	X
Gait speed	Gait speed four-meter walking test	X	X	X	X
Weight loss	The Göteborg Quality of Life Instrument	X	X	X	X
Cognition	Mini Mental State Exam (MMSE)	X		X	X
Visual impairment	KM visual acuity chart	X		X	X
Self-rated health	SF 36 (a single question)	X	X	X	X
Illness	CIRS-G	X		X	X
Symptoms	The Göteborg Quality of Life Instrument	X	X	X	X
Depression	GDS 20	X	X	X	X
Activities of daily living	The ADL staircase	X	X	X	X

**Secondary Outcomes**	**Measurement**				

Health-related quality of life	EQ5D	X	X	X	X
Life satisfaction	Fugl-Meyer -- LiSat	X	X	X	X
Assistive technology and accessibility	Questionnaire	X	X	X	X
Participation/Leisure activities	Questionnaire	X	X	X	X
Social support	Questionnaire	X	X	X	X
Social network	Questionnaire	X		X	X
Falls	Questionnaire	X	X	X	X
Fear of falling	FES-I	X	X	X	X
Health care Consumption	Register data				

### Procedure

Eligible persons for the study were drawn from official registers of all persons over 80 years of age in the two municipalities. Equal numbers from the two municipalities were listed in random order. The persons were included in the sample consecutively using the simple random sampling chart until the intended sample size was reached. Invitation letters were then sent to all persons in the sample (n = 2031) asking them to participate in the study. The letter described the study, how it would be conducted and what would be expected of those consenting to participate. The letter stressed the fact that participation was voluntary. The letter was followed up by a telephone call about 1-2 weeks later. 365 persons out of 2031 persons were either non-eligible (n = 147) or not traceable (n = 218). The remaining persons (n = 1666) was then informed verbally about the study and given the opportunity to ask questions if anything was unclear. They were also asked personally if they would like to participate, while again stressing that this was voluntary. 1120 persons out of the 1666 persons were unwilling or unable to participate (no interest n = 936, lack of time n = 116, not having the strength n = 68). For the 546 persons who consented to participate, a time for the first visit, when they also would have to hand in their written consent, was decided.

After a baseline interview, 491 persons were found to fulfil the study criteria and were randomised to one of the three study arms by the use of sealed opaque envelopes. If eligible persons lived together, they were always allocated to the same intervention group.

One hundred and fourteen persons were allocated to the control group, 178 to the preventive home visits and 199 to the senior meetings. Four persons allocated to preventive home visit and 23 to the senior meetings withdrew their consent because they did not want to participate in the allocated intervention. Further, 5 persons participating in senior meetings fell ill. Thus, 459 persons were included in the study, 114 in the control group, 174 in the preventive home visits and 171 in the senior meetings.

The study was preceded by a pilot study with the purpose of testing intervention, inclusion criteria and logistics in which the elderly themselves, the professionals and the research assistants were involved. The research assistants were occupational therapists, physiotherapists or nurses. They were trained and inter-rater reliability was tested. To enhance the quality of outcome measurements, study protocol meetings were held throughout the study. Further, another research assistant who had not conducted the interview was responsible for the data entry at baseline. These data was verified in a second step by yet another of the research assistants. Data entry at follow-ups was in charge of the interviewers. These data were verified in a second step by another research assistant who had not conducted the interview.

All participants received a first visit that comprised an interview, assessment and observation. The first visit, i.e. the baseline interview, was performed in the participant's home by research assistants well trained in interviewing, assessing and observing according to the guidelines for the different outcome measurements. Follow-up data were also collected in the participant's home by well-trained research assistants. The research assistants that assessed the outcomes were not involved in the intervention and were blind to group assignment.

**Figure 1:**
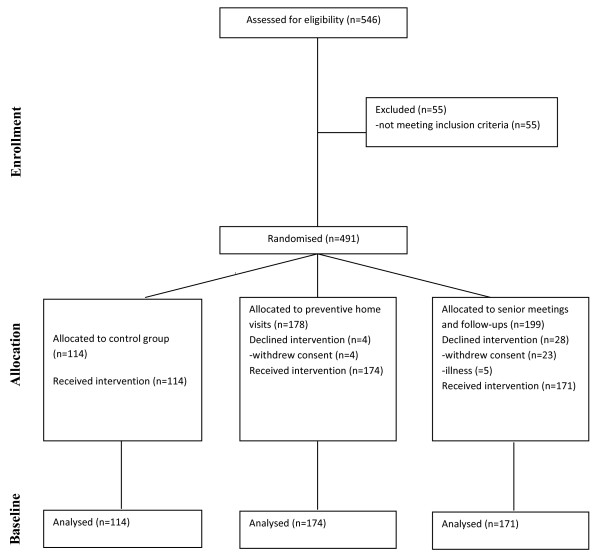
**Flow chart**. Flowchart throughout enrollment, allocation and baseline.

Follow-up data were collected in all groups at 3 months, 1 year and 2 years after intervention (see figure 1).

### Statistical analysis and power calculations

The calculations will be based on the expected relative change in time, i.e. we assume that the intervention group "Senior meetings and a follow-up home visit" will change slightly or not at all in functional status; that the intervention group "Preventive home visit" will change by 15% in relation to the intervention group with Senior meetings while the "Control group" will deteriorate 20% more than the intervention group "Preventive home visit". Power calculation ensures that, if the hypotheses are true, we will be able to discover a difference of at least 15% between the intervention groups and a difference of at least 20% between the intervention groups and the control group.

Since a level of significance of alpha = 0.05 and a power of 80% are required to compare the intervention groups in a two-sided test, at least 112 persons will be required in each intervention group to be able to observe a difference of at least 15% between the groups. A comparison between the control group and the intervention groups will require 72 persons in the control group, if we assume a difference of at least 20%. A total of about 300 persons will be required.

However, to allow for drop-outs, it was estimated that a total of about 450 persons would need to be included in the study.

The analyses will be made on the basis of the intention-to-treat principle, which means that the participants will be analysed on the basis of the group to which they have been randomised [34]. Both descriptive and analytical statistics will be used to compare the groups as well as for analyses measuring changes over time. Non-parametric statistics will be used in all cases where ordinal data are analysed. Otherwise parametric statistics will be used.

### Qualitative methods

A qualitative approach [35] is used to gain an understanding of the elderly person's experiences of the intervention and its effects. Both individual interviews and focus groups are conducted with the persons involved in the study. This will enable analyses of individual differences and similarities in the groups that participate in the study, as well as differences and similarities between the interventions groups.

*The focus group methodology *[36,37], which is a form of group discussion, especially utilises group interaction. Focus groups are described as providing an insight into the world of the participants, examining the target group's shared understanding of everyday life, language, and culture. Focus group methodology is regarded as especially useful when the experiences of individuals with limited power and influence are explored. Being among others in a non-threatening and permissive environment with people who share many feelings and experiences can provide them with the power to express their perspective.

*Individual interviews *[35], which may be characterised as 'in-depth' interviews, have been carried out with the elderly participating in the study. The interviews aimed to elicit the individual experiences of participating in the health-promoting and preventive intervention programme.

*Both the focus group and the individual interviews *have been audio-taped, transcribed verbatim and analysed, identifying patterns and themes in the interview statements. The qualitative data will enable an analysis of the similarities and differences between the participating elderly persons' perceptions and experiences on an individual as well as group level. This qualitative study may contribute important knowledge that will improve out understanding of the effects of the intervention.

### Time plan of the study

The study started in November 2007. The inclusion of 491 persons was completed in November 2008. The intervention started in January 2008 and was completed by the end of December 2008. The 3-month follow-up was completed in March 2009. The one-year follow-up started in January 2009 and will be completed by the end of January 2010. The individual interviews and focus groups started during October 2008 and are expected to be completed in 2010. The final follow-ups are expected to be completed by the beginning of 2011.

### Baseline characteristics

There were no statistically significant differences between the control group and the interventions groups in baseline characteristics in terms of demographic and frailty indicators, see table 2 and table 3. The median age of the participants was 86 years (range 80-97) in the control group, 86 years (range 80-94) in the preventive home visit intervention and 85 years (range 80-94) in the senior meetings and follow-up intervention.

**Table 2:** Baseline characteristics of study participants

Characteristics	Control group	Preventive home visits	Senior meetings and follow-up	P-value
	n = 114	n = 174	n = 171	
	%	%	%	
Female	61	64	66	0,63
Living alone	48	57	60	0,10
Academic education	22	23	19	0,69
Self-rated health (excellent/verygood/good)	79	80	83	0,63

**Table 3:** Frailty indicators of study participants

Frailty indicators	Control group	Preventive home visits	Senior meetings and follow-up	P-value
	n = 144	n = 174	n = 171	
	%	%	%	
Weakness	11	10	6	0,285
Fatigue	36	39	42	0,633
Physical activity	29	22	21	0,278
Weight loss	6	8	5	0,698
Gait speed	11	15	9	0,248
Poor balance	18	17	12	0,371
Visual impairment	62	63	63	0,98

## Discussion

The three-armed study "Elderly in the risk zone" is designed to evaluate if multi-dimensional and multi-professional educational senior meetings are more effective than preventive home visits, and if it is possible to prevent or delay deterioration if an intervention is made when the persons are not so frail. The fact that this study has both an explorative and experimental design, which facilitates a multi-facetted knowledge production, may be considered its major strength. In addition, the combination of qualitative and quantitative methods could maximise the ability to bring different strengths together and provide a unique opportunity to see what is in the "black box", i.e. to generate unexpected or unpredictable knowledge[38].

Through a power calculation it was estimated that a total of about 450 persons needed to be included in the study to allow for drop-outs. To reach the intended sample size, invitation letters was sent to all persons without home care in two municipalities of the city of Gothenburg. One weakness was the large amount of primary drop-outs, which may affect the generalisation of the study [34]. Studies have shown that the older the persons are, the higher the number of drop outs, leading to a risk of selectiveness [39]. In this study a large proportion declined to participate because they were not interested or had no time. It also became evident at the educational senior meetings as well as at the preventive home visits that these elderly persons did not regard themselves as target groups for aged care interventions and had been hesitant about participating in the study. This is a methodological problem for studies whose target group are independent elderly persons.

Although few drop outs are desirable, it is important to ask whether those who participated in the study are representative of the population of interest. In our study, the baseline characteristics indicated that about 80% of the participants experienced good/very good or excellent health. Nevertheless, the frailty indicators reveal that the participants experienced different degrees of frailty. Approximately 40% of the participants experienced fatigue, 60% were visually impaired, and 22-36% reported a low level of physical activity. This can be seen as evidence for the capture of the target group. However, according to statistical theories, proper randomisation should guarantee that the groups are comparable in baseline characteristics and ensure that differences between groups are due to trial effects [34]. In this study there were no statistically significant differences between the control group and the interventions groups in baseline characteristics in terms of demographic and frailty indicators, showing that randomisation was properly performed.

To enhance its quality, the study was preceded by a pilot study with the purpose of testing intervention, inclusion criteria and logistics by involving pensioners' representatives and responsible professionals in the municipality in the different phases of the planning. Meetings were held with pensioners' representatives to discuss the content and the extent of the interview. Pilot interviews were conducted in order to test the questions and the outcome measurements. Their experiences of the interview were discussed in the groups, which led to a clarification of questions and removal or substitution of outcome measurements.

Discussions were also held with the representatives about the content of the interventions and the educational material used in the interventions. The fact that the elderly themselves were involved in planning and developing the programme in close cooperation with the professionals should ensure that it will meet their needs [40]. This might mean that the intervention itself is more likely to be effective.

Furthermore, the researchers involved responsible professionals in the municipality in the implementation of the interventions. The professionals participated in the development of the intervention, and meetings were held in order to discuss the intervention, inclusion criteria and logistics. The intention was that research and ordinary field activities should get closer by working hand in hand throughout the project and inspire each other. Nevertheless, we were aware of the importance of making a distinction between those involved in the intervention and those conducting research on the intervention. Accordingly, the research assistants in this study have not been involved in the intervention [41]. This also facilitated the blinding of the study.

When we set up both the research group and the group of professionals carrying out the interventions, we intentionally strove for a multi-professional composition. This was done in order to ensure the multi-dimensionality of the study, both in performing the interventions and in interpreting the results of the study. The researchers involved in the study represented occupational therapy, physiotherapy, medicine, nursing, social work and health economist. The data collectors/assessors were occupational therapists, physiotherapists or nurses. These different professional backgrounds may affect the measurement quality. However, they were all trained, and inter-rater reliability was tested. In addition, study protocol meetings were held throughout the study period in order to identify and deal with unexpected problems. To further enhance the quality of the study, the outcome measurements were selected very carefully to make sure that they had clear psychometric properties, i.e. were valid and reliable for the target group and measured/covered the different components of the concept of frailty.

In summary, the design of the study, the randomisation procedure, outcome measurements and the study protocol meetings should ensure the quality of the study. Furthermore, the multi-dimensionality of the intervention, the involvement of both the professionals and the senior citizens in the planning of the intervention should have the potential to effectively target the heterogeneous needs of the elderly.

## Competing interests

The authors declare that they have no competing interests.

## Authors' contributions

SDI led the research design, intervention and implementation of the study and was primary author of the manuscript. AKE participated in the research design and intervention of the study. SL participated in the research design and implementation of the study. GGH participated in the research design and contributed to the questionnaire regarding activity and assistive technology. AD participated in formulating the aim of the study and composed the social outcome measurements. KE and KW compiled the outcome measurements covering the concept of frailty. LZ and AKW contributed to the questionnaire regarding physical activity. All authors contributed to the writing and review of the manuscript and approved the final manuscript.

## Pre-publication history

The pre-publication history for this paper can be accessed here:

http://www.biomedcentral.com/1471-2318/10/27/prepub
